# Arbuscular mycorrhizal fungi change root morphology and nutrient use efficiency in the tree legume *Mimosa scabrella*

**DOI:** 10.1007/s00572-026-01260-9

**Published:** 2026-04-14

**Authors:** Silmar Primieri, Murilo Dalla Costa, Tássio Dresch Rech, Marlise Nara Ciotta, Sidney L. Stürmer

**Affiliations:** 1 Campus Lages, Federal Institute of Education, Science and Technology of Santa Catarina (IFSC), Lages, SC 88509-640 Brazil; 2 Experiment Station of Lages, Santa Catarina State Agricultural Research and Rural Extension Agency (EPAGRI), P.O. Box 181, Lages, SC 88502-970 Brazil; 3 Departamento de Ciências Naturais, Universidade Regional de Blumenau, R. Antônio da Veiga 140, Blumenau, Santa Catarina 89030-903 Brazil

**Keywords:** Leguminosae, *Mimosa scabrella*, Phosphorus fertilization, Symbiosis, Arbuscular mycorrhizal fungi, Mycorrhizal responsiveness

## Abstract

*Mimosa scabrella* is a leguminous tree native to southern Brazil with high economic potential as a source of timber and biomass. This plant interacts simultaneously with multiple microbial partners in symbiosis, including arbuscular mycorrhizal fungi (AMF). However, the extent of the fungal role and the impact of different AMF species on growth and nutrition remain unclear. This study aimed to evaluate the effects of three AMF species on the growth, nutrient uptake, and root traits of *M. scabrella* seedlings under varying phosphorus (P) levels. Seedlings were cultivated in a greenhouse for 60 days in sterile sand-soil inoculated with *Acaulospora colombiana* (AC), *A. morrowiae* (AM), and *Rhizophagus clarus* (RC) across five P levels (0; 20; 40; 80; or 160 mg dm⁻³). AMF inoculation strongly enhanced plant growth and nutrient acquisition across P levels, with high levels of mycorrhizal colonization observed even at the highest P concentrations. Biomass production varied among AMF species: AC and RC increased growth 11.6x and 11.7x, respectively, while *A. morrowiae* promoted a 5.1x increase. Inoculated plants showed reduced specific root length and greater average root diameter (except at 160 mg P dm⁻³), suggesting a potential shift in resource allocation. Concentrations of N, P, and K, as well as nutrient use efficiency (NUE), were consistently elevated in inoculated seedlings, though increases in NUE may partly reflect overall plant size. Despite all AMF species yielding positive results, AC and RC were notably more effective than AM. These findings highlight the importance of considering AMF host preference and local soil conditions in optimizing plant-AMF symbioses. Such optimizations are crucial for enhancing practices in forestry, agriculture, and environmental reclamation. While our results indicate high mycorrhizal responsiveness, the co-inoculation with *Burkholderia* spp. necessitates caution in inferring true mycorrhizal dependence, and the functional significance of high colonization at elevated P requires further investigation.

**Supplementary Information:**

The online version contains supplementary material available at 10.1007/s00572-026-01260-9.

## Introduction

*Mimosa scabrella* Benth (commonly known as Bracatinga) naturally occurs in association with the Araucaria forest in Southern Brazil (Zangalli et al. 2023) where it can form very dense and almost pure stands (Reitz et al. 1978). *Mimosa scabrella* holds significant ecological and economic importance in the region as it is commonly used as an energy source (Barth 2004; Mazuchowski et al. 2014) and plays a crucial role in producing high-value honeydew honey for small farmers. This honey is derived from the melate produced by mealybugs that colonize the stems of *M. scabrella*. The melate, a compound consisting of various sugars and other substances, is harvested by bees to produce honeydew honey as an alternative food source after the flowering period of plants (Müller 2011). *M. scabrella* is an important species for restoring areas after silvicultural use and for reclaiminig mining sites (Camargo et al. 2016; Citadini-Zanette et al. 2017) largely due to its nitrogen-fixing ability (Franco and Faria 1997; Primieri et al. 2016).

As a pioneer species during secondary forest succession, this plant thrives in nutrient-poor environments (Mazuchowski et al. 2014) and their growth and nutrition are enhanced when associated with symbiotic microorganisms (Lammel et al. 2015; Camargo et al. 2016; Primieri et al. 2016, 2021). The association between *M. scabrella* and arbuscular mycorrhizal (AM) fungi was first reported by (Andrade et al. 2000) who observed hypha and vesicles colonizing roots under natural conditions. Melloni et al. (2003) recovered six AM fungal species from natural stands of *M. scabrella.* (Lammel et al. 2015) and Camargo et al. (2016) demonstrated that *M. scabrella* is highly responsive to AM fungi inoculation with biomass production increasings of 102–513% when inoculated with various species of AMF. These experiments resulted in increases not only in plant biomass, but also on P and trace elements (Ar, Cu, Zn, Cr, Cd) contents and accumulation, and number and weight of nodules. (Primieri et al. 2021) showed that inoculation with *Rhizophagus intraradices* improved plant biomass accumulation of *M scabrella*, and this effect was further amplified when plants were co-inoculated with the nitrogen-fixing bacterium *Burkholderia* in a tripartite association with the host plant, resulting in a synergistic growth response. These findings align with broader patterns revealed by meta-analyses, which demonstrate that synergistic benefits of co-inoculation with AMF and rhizobia are characteristic of perennial legumes, whereas annual species typically exhibit only additive or non-synergistic responses (Primieri et al. 2022; Priyadarshini et al. 2022).

However, the effect of AM fungi inoculation on *M. scabrella* responsiveness have been studied in soils with low P available and under only one P level. This condition can significantly influence the establishment and benefits of the mycorrhizal association as a gradient of phosphorus availability plays a critical role in determining the extent of the benefits conferred by AM fungi (Smith and Read 2008). As phosphorus levels increase, the extent of the mycorrhizal symbiosis can vary among different AM fungal genera (Treseder et al. 2002), thereby influencing plant growth, nutrient use efficiency, and root.

Inoculation with AM has a remarkable potential to enhance nutrient use efficiency and alter plant root morphology, particularly in phosphorus-deficient soils. A meta-analysis conducted by (Schütz et al. 2018) strengthened the critical role of AM fungi in improving P and N use efficiency, with results showing significant yield increases of over 40% under optimal soil phosphorus conditions. Nutrient use efficiency is indirectly affected by changes in root morphology and physiology that are induced by AM fungal colonization. These changes include an increase in the root volume and total root length, as well as the development of finer and more highly branched roots, that increases the area explored by the roots (Chandrasekaran 2022). Moreover, AM colonization stimulates the production of root exudates that promote the solubilization of less available nutrients in the soil, such as phosphorus and micronutrients, while also attracting beneficial microorganisms that further support nutrient acquisition (Bhupenchandra et al. 2024). These benefits are largely due to traits associated with the large hyphal network produced by these fungi, the intraradical and extraradical mycelium, that ultimately are responsible for several plant- and ecosystem-level processes related to AM fungi (Antunes et al. 2025).

Considering that the combination of AM fungal species, plant host and soil P content is important to determine the outcome of the mycorrhizal symbiosis, we aimed to investigate how inoculation with three species of AM fungi influences growth, nutrient uptake and root architecture of *Mimosa scabrella* seedlings under different levels of soil phosphorus. We calculated the nutrient use efficiency index for eight nutrients, mycorrhizal efficiency and responsiveness. Previous findings show that *M. scabrella* is highly responsive to mycorrhiza (Lammel et al. 2015; Camargo et al. 2016; Primieri et al. 2021), therefore we predict that inoculation with AM fungi increase plant biomass accumulation of *M. scabrella*, independently of P level in the soil. Considering that AM fungi inoculation have been shown to increase nutrient use efficiency for P and N (Schütz et al. 2018) and change root architecture (Chandrasekaran 2022), we predict that AM fungi modifies root architecture and nutrient use efficiency in *M. scabrella*.

## Materials and methods

### Biological material

Seeds were collected from a single *M. scabrella* plant in Urupema, Santa Catarina state, southern Brazil (27°57’41.75 “S and 49°50’40.90"W) and stored at 2 to 5 °C until germination.

The AM fungal isolates were obtained from the International Culture Collection of Glomeromycota (CICG at http://www.furb.br/cicg; Universidade Regional de Blumenau) and included *Acaulospora colombiana* SCT115A, *A. morrowiae* SCT400A and *Rhizophagus clarus* SCT720A. Inoculum of each isolate consisted of spores, hyphal fragments, and colonized root pieces in a soil: sand growth medium. To bulk up the soil inoculum of each fungal isolate for the experiment, inoculum from stock cultures was diluted (10%) with a sterile substrate consisting of a 1:1 mixture (vol/vol) of a silt loam soil and quartzite sand in 1.5 kg plastic pots. Seeds of *Urochloa brizantha* Moench were added to each pot and plants were grown in a greenhouse. After four months, watering was ceased, the plant tops removed and discarded, and the substrate with roots balls was chopped, homogenized, and stored at 4° C until used in the plant growth experiment. Pots containing only the sterile substrate were also established and used in the non-mycorrhizal control treatment.

Two selected strains of nitrogen-fixing bacteria, isolated from *M. scabrella* nodules, both identified as belonging to the *Burkholderia* genus (Primieri et al. 2016), were used to inoculate the seedlings in the experiment. The sequences are available from GenBank (www.ncbi.nlm.nih.gov/genbank), accession numbers KP126630 and KP126654.

### Experimental design and growth conditions

A plant growth experiment was conducted using 4.5 L plastic pots filled with Inceptisol. The soil was sterilized twice by autoclaving at 121 °C for 1 h with a 24 h interval between sterilization cycle. The soil had the following properties: 2.5% organic matter, pH 4.7, 6.1 mg/ dm³ extractable P, 32 mg/ dm³ K, 2.6 cmolc/ dm³ Ca, 0.7 cmolc/ dm³ Mg, and 0.7 cmolc/ dm³ Al. After sterilization, calcitic limestone was added to adjust the soil pH to 5.5, and the potassium levels were adjusted by applying 65 kg K₂O/ha (equivalent to 62 mg KCl/dm³), following the fertilization recommendations for forest species (CQFS RS/SC, 2004). These amendments were made one week before sowing.

The experiment with *M. scabrella* and AM fungi followed a factorial, completely randomized design, with six replicates per treatment combination. Treatments consisted of four inoculation treatments (*A. colombiana*, *A. morrowiae*,* R. clarus*, and non-mycorrhizal control) and five levels of P (0, 20, 40, 80, and 160 mg dm^− 3^) added to the soil. The level of phosphorus in soil were established according to Soil Chemistry and Fertility Commission of Rio Grande do Sul and Santa Catarina (CQFS-RS/SC 2004). In addition to the control treatment (0 mg dm^− 3^), P levels were established by adding Na_2_HPO_4_ representing the rate recommended for the culture (20 mg dm^− 3^), high availability (40 mg dm^− 3^) and very high availability of P (80, and 160 mg dm^− 3^). Experimental units were distributed randomly in a greenhouse set at 28 ± 2 °C at the Experimental Station of Lages - Santa Catarina State Agricultural Research and Rural Extension Agency (EPAGRI).

Pots assigned to mycorrhizal treatments were inoculated with 25 g inoculum per experimental unit and consisted of adding soil inoculum of each isolate as a layer 1 cm below the surface of the substrate. Pots assigned to the non-mycorrhizal control received 25 g of the non-mycorrhizal inoculum produced in the same conditions used to bulk up the fungal isolates inoculum. *Burkholderia* spp. strains were cultured in yeast extract mannitol (YM) broth (Vincent 1970), incubated at 28 °C in an incubator shaker, and 1mL inoculated in all pots, containing approximately 10^9^ CFU mL^− 1^ (Hungria and Araújo 1994). Inoculation was performed 7 days after transplanting the seedlings, and a sterile YM was added to the control.

*Mimosa scabrella s*eeds were surface sterilized with 96% ethanol for 1 min, 3% sodium hypochlorite for 5 min and rinsed in distilled water. To overcome dormancy, seeds were immersed in hot water (80 °C) for 5 min and left soaking at room temperature for 18 h. Two seeds were sown in each 3.6 L pot (positioned just above the inoculum layer for AM fungal treatments) and thinned to one seedling after emergence. All pots were watered to capacity with distilled water daily.

### Harvest and response variables

After grown for 60 days in a greenhouse, plants were cut at the soil line, and shoots were placed in paper bags and dried in a forced-air oven (65–70 °C) to a constant weight for the determination of shoot dry biomass. The roots were carefully washed under water to remove soil debris and stored in 50% ethanol.

The entire root system was scanned using the WinRhizo Pro (2009) scanning software (Regent Instruments Canada Inc.) system with the Epson Expression 10,000 XL scanner. To scan, the roots were removed from 50% ethanol and immersed in water in a transparent tray. Data were collected for each replicate for the following root traits: total root length (m plant^− 1^), surface area (cm^2^ plant^− 1^), average diameter (mm), volume occupied in soil (cm^3^ plant^− 1^), number of crossings and number of tips. After scanning, the roots were forced-air oven dried (65–80 C) and weighed to obtain the total root dry weight. To explore the nutrient foraging strategy of *M. scabrella*, the specific root length (SRL expressed as m g^− 1^ root dry weight) was calculated by dividing the total root length by the total root dry weight (Freschet and Roumet 2017; Wen et al. 2019).

Prior to drying, root samples were cut into 1 cm segments and placed in tissue cassettes (HistoPrep OmniSette; Fisher Scientific Healthcare, Pittsburgh, PA, USA) for assessment of mycorrhizal colonization. Samples were immersed in 10% KOH for 1 h at 80 °C, rinsed under tap water, acidified with 1% HCl for 20 min, and then stained with ink-vinegar solution (Vierheilig et al. 1998) using blue ink 5% Sheaffer (#728-8563-BLK) with 5% acetic acid for 10 min at 90 °C. The stained roots were placed in lactoglycerol overnight to remove the residual stain. The roots were then placed on microscope slides and inspected for fungal features (i.e. hyphae, vesicles and arbuscular colonization). The percentage of mycorrhizal root colonization was measured by the gridline intersect method (McGonigle et al. 1990) at 200 x magnification obtained through the number of colonized segments in relation to the total analyzed (100 spots per sample).

Shoot samples were analyzed for N, P, K, Mg, Ca, Fe, Mn Zn, and B according to the standard method described by (Tedesco et al. 1995). The nutrient concentration (%) was multiplied by the total dry weight to estimate the content of each nutrient in the plant. The results were also used to calculate the nutrient use efficiency index (NUE) of each nutrient, calculated using Eq. (1), where $\:TDW$ is the total dry weight and “X” is the nutrient content (i.e. phosphorus, nitrogen, calcium, iron, magnesium etc.) expressed as g plant^− 1^.

1 $$\:NUE=\frac{\left(TDW\right)^2}{"X"\:content\:\left(g\right)}$$

Using total dry weight, we calculated the mycorrhizal efficiency (ME) Eq. (2a) and mycorrhizal responsiveness (MR) as Eq. (2b): where $\:{TDW}_{M}$ is the total dry weight of treatment with mycorrhiza and $\:{TDW}_{NM}$ is the total dry matter of treatment without mycorrhiza, multiplied by 100, expressed as a percentage of the dry biomass of inoculated plants (Janos 2007; Plenchette et al. 1983).

2 $$\begin{array}{c}\begin{array}{c}a\;\;\;\;\;\;\;ME=\frac{{TDW}_M-{TDW}_{NM}}{{TDW}_{NM}}\ast100\\b\;\;\;\;\;\;\;MR=\frac{{TDW}_M-{TDW}_{NM}}{{TDW}_M}\ast100\end{array}\end{array}\\$$

### Statistical analysis

All data were previously checked for normality and homogeneity of variances and transformed when necessary. A fully factorial analysis of variance (ANOVA) and post hoc tests were conducted using Tukey test (*p* < 0,05). Quantitative data were subjected to a regression analysis, and the regression coefficients were analyzed using Student’s t-test. Correlation analysis was carried out including all root measurements (i.e., total root length, surface area, length per volume, volume occupied in soil, number of crossings and number of tips), root dry biomass and shoot dry biomass. We also correlated specific root length (SRL) and shoot dry biomass with total AM fungal colonization. Statistical analyses were performed using R software (R Core Team 2018).

## Results

### Plant growth

**Fig. 1:**
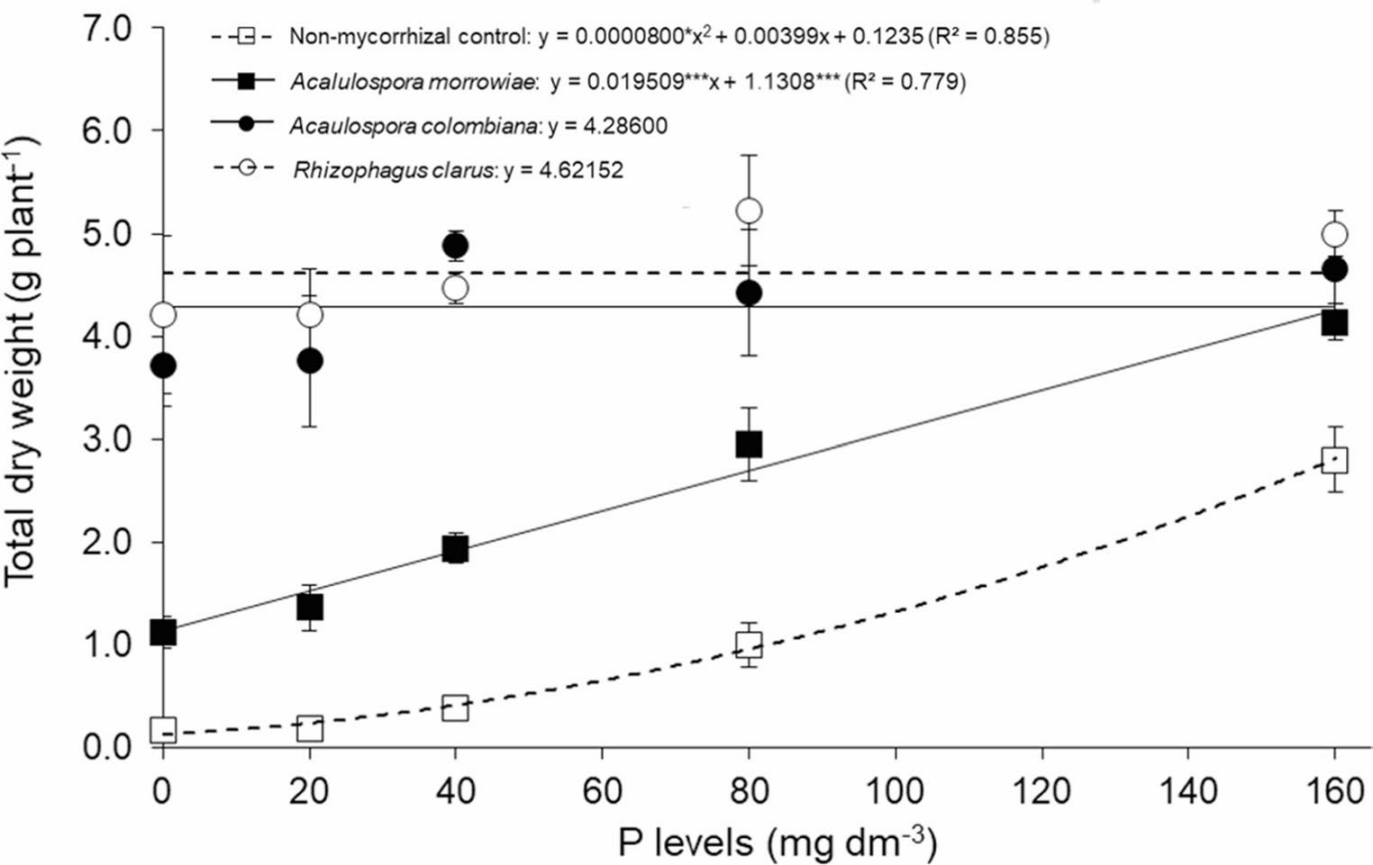
Total dry weight (g) of *Mimosa scabrella* inoculated with *Acaulospora morrowiae*, *Acaulospora colombiana* and *Rhizophagus clarus* and non-mycorrhizal control under different P levels. Averages were obtained from 6 replicates and error bars represent the standard error of the mean. Horizontal lines are non-significant regression. Asterisks represent significant differences as calculated by ANOVA (*p < 0.05, **p < 0.01, ***p < 0.001)

*Mimosa scabrella* seedlings responded to inoculation with all AMF, showing different magnitudes of growth depending on the AMF inoculated and P levels (Fig. 1). Considering all P levels, the increase of shoot dry weight was 11.6x, 11.7x and 5.1x higher when inoculated with *Acaulospora colombiana* (AC), *Rhizophagus clarus* (RC) and *Acaulospora morrowiae* (AM), respectively, compared to non-mycorrhizal control (Fig. 1). A quadratic regression model successfully fits the growth response of *M. scabrella*, in terms of phosphorus supply for all treatments. However, there was no difference for dry weight when *M. scabrella* seedlings were inoculated with AC and RC, independent of P levels.

In general, shoot dry weight of *M. scabrella* seedlings associated with AMF was larger compared to non-mycorrhizal control for all P levels. However, as the P levels in the substrate increased, differences between mycorrhizal and non-mycorrhizal plants decreased. For example, the total dry weight of *M. scabrella* seedlings inoculated with AC at 20 mg P dm^− 3^ was 21x higher compared to the control while at 160 mg P dm^− 3^, this difference decreased to 1.6x.

AMF inoculation significantly increased *M. scabrella* biomass, but the results varied depending on the AMF species used. Plants inoculated with *Acaulospora morrowiae* at 0 mg P dm⁻³ increased dry biomass weight by 6.6x compared to the non-mycorrhizal control, while inoculation with AC and RC resulted dry biomass weight 21.8x and 24.7x higher compared to non-mycorrhizal control, respectively. However, at 160 mg P dm⁻³, these differences between AMF treatments were no longer evident (Fig. 1). At 160 mg P dm⁻³, AMF inoculated plants showed substantial growth, with biomass weight 27x greater than the non-mycorrhizal control at 0 mg P dm⁻³.

### Host colonization and responsiveness to mycorrhizae

Roots of *Mimosa scabrella* were colonized by all AM fungi, and no evidence of mycorrhizal colonization was observed in non-mycorrhizal control plants. Overall, root colonization by *A. morrowiae* was lower (74%) compared to *A. colombiana* (84%) and *R. clarus* (91%). These results were more evident in the treatment without P added, where the levels of mycorrhizal colonization were 39.5% higher in *A. colombiana* and *R. clarus* than in *A. morrowiae.* The magnitude of these differences decreased with the addition of phosphorus mycorrhizal colonization was not significantly different among AM fungal isolates at 160 mg P dm^− 3^ (Table 1). Colonization by AC tended to increase as P levels increased while high levels of colonization were detected for AC and RC at 0 mg P dm^− 2^ and remained relatively stable with higher levels of P soil (Table 1).

**Table 1:** Total mycorrhizal colonization (%) of *M. scabrella* roots inoculated with the arbuscular mycorrhizal (AM) fungus *Acaulospora morrowiae*, *Acaulospora colombiana* and *Rhizophagus clarus* cultivated at different levels of P. Means followed by the same lower case letters within columns and same upper case within lines are not statistically different as determined by the Tukey test (*p* < 0.05)

	*P* supply (mg *P* dm^− 3^)				
	0	20	40	80	160
*A. morrowiae*	66.7 bCD	77 bBC	55.2 bD	79 abAB	91.3 aA
*A. colombiana*	92.8 aA	75.2 bB	80.3 aB	78.2 bB	93.6 aA
*R. clarus*	93.3 aA	92.8 aA	86.8 aA	89.5 aA	93 aA
C.V.	13%				

**Fig. 2:**
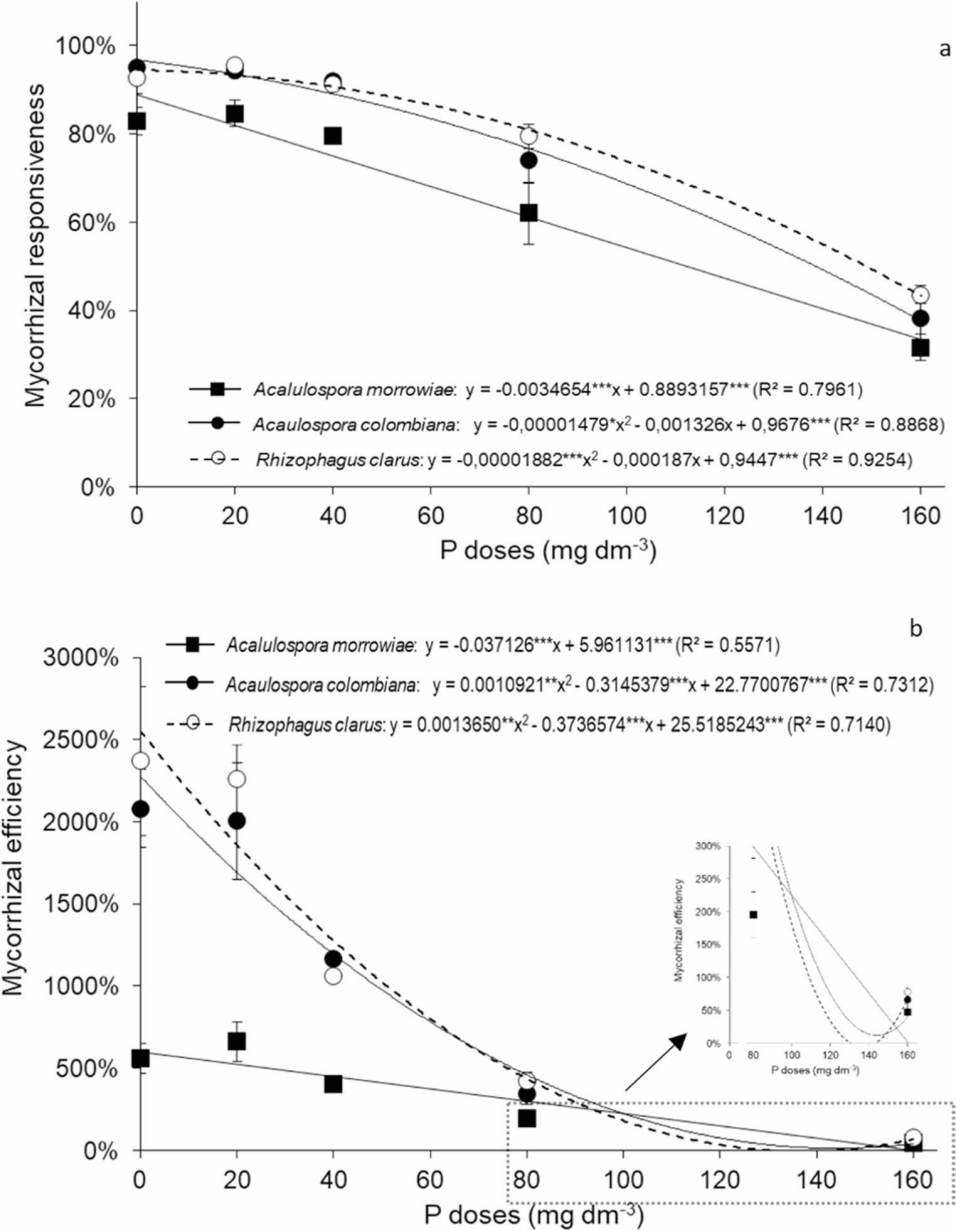
Mycorrhizal responsiveness (**a**) and mycorrhizal efficiency (**b**) from *Mimosa scabrella* seedlings inoculated with *A. colombiana*,* R. clarus* and *A. morrowiae*, cultivated under different P levels. Averages were obtained from 6 replicates and error bars represent the standard error of the mean. Asterisks represent significant differences as calculated by ANOVA (*p < 0.05, **p < 0.01, ***p < 0.001)

Mycorrhizal responsiveness decreased as soil P levels increased, regardless of AM fungal species (Fig. 2A). Mycorrhizal responsiveness (MR) was higher than 90% in seedlings inoculated with *A. colombiana* and *R. clarus* at 0 to 40 mg P dm^− 3^, decreasing to 70% at 80 mg P dm^− 3^ and 40% at 160 mg P dm^− 3^ (Fig. 2A). Plants inoculated with *A. morrowiae* showed MR values between 80% and 84% at 0 to 40 mg P dm⁻³, which decreased to 62% at 80 mg P dm⁻³ and to 31% at 160 mg P dm⁻³.

Although AM fungal inoculation promoted plant growth, the mycorrhizal efficiency was 272% and 324% higher for *A. colombiana* and *R. clarus*, respectively, compared with *A. morrowiae* at 0 mg P dm^− 3^ (Fig. 2B). The magnitude of the mycorrhizal efficiency decreased with increasing P supplementation, and at 160 mg P dm^− 3^, the mycorrhizal efficiency of *A. colombiana* and *R. clarus* was 39% and 65% higher, respectively, compared to *A. morrowiae*, respectively.

### Root traits response

Root traits - including root length, projected area, surface area, root volume, and number of tips, were all significantly correlated with one another, as well as with total plant biomass (*p* < 0.001) (Supplementary Material Table S1). Inoculation with AM fungi significantly increased all root traits in treatments at 0, 20, 40, and 80 mg P dm^− 3^, but not at160 mg P dm^− 3^ (Supplementary Material Fig. S1).

**Fig. 3:**
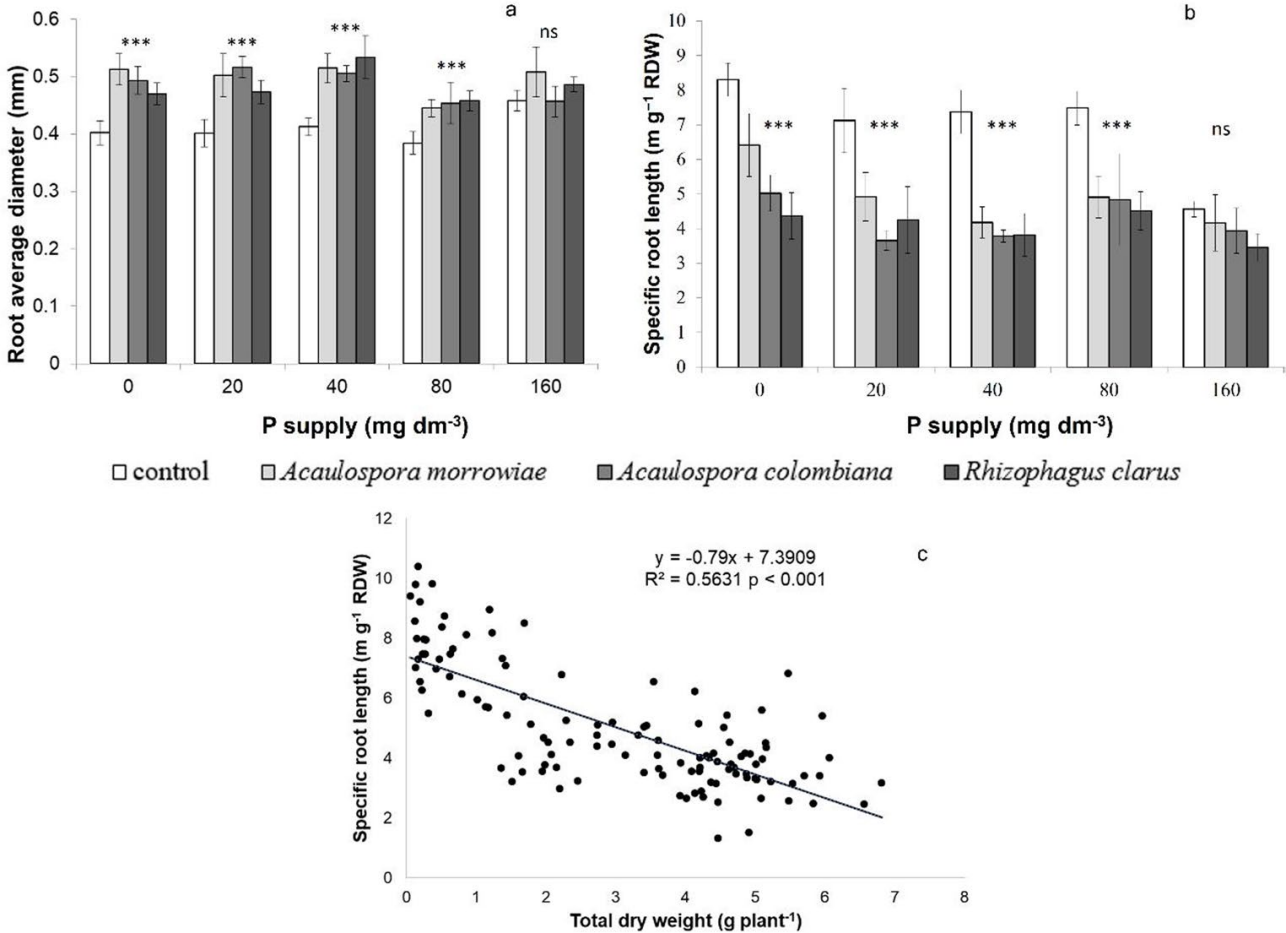
Root average diameter (**a**) and specific root length (**b**) of *Mimosa scabrella* roots inoculated with *Acaulospora morrowiae*, *Acaulospora colombiana* and *Rhizophagus clarus* and non-mycorrhizal control under different P levels. Correlation between specific root length (m g^− 1^ RDW) and total dry weight (g plant^− 1^) (**c**). Bar represents means (*n* = 6), and error bars represent the standard error. Asterisks represent significant differences (* *P* < 0.05). The trendline has been added to Fig. 3C. Asterisks represent significant differences as calculated by ANOVA (***p < 0.001; ns – non significant)

Inoculation with AM fungi significantly increased root average diameter (*p* < 0.001) by approximately 1 mm in treatments with 0, 20, 40, and 80 mg P dm^− 3^ (Fig. 3A). Conversely, AM fungi inoculation significantly reduced specific root length (SRL) by approximately 37% compared to non-inoculated plants under the same phosphorus levels (*p* < 0.001) (Fig. 3B). However, no differences in the root average diameter or SRL were observed at 160 mg P dm⁻³. We found a significant negative correlation between the total dry weight and SRL (r² = 0.56; *p* < 0.001; Fig. 3C).

### Effect of AMF inoculation on nutrient uptake

**Fig. 4:**
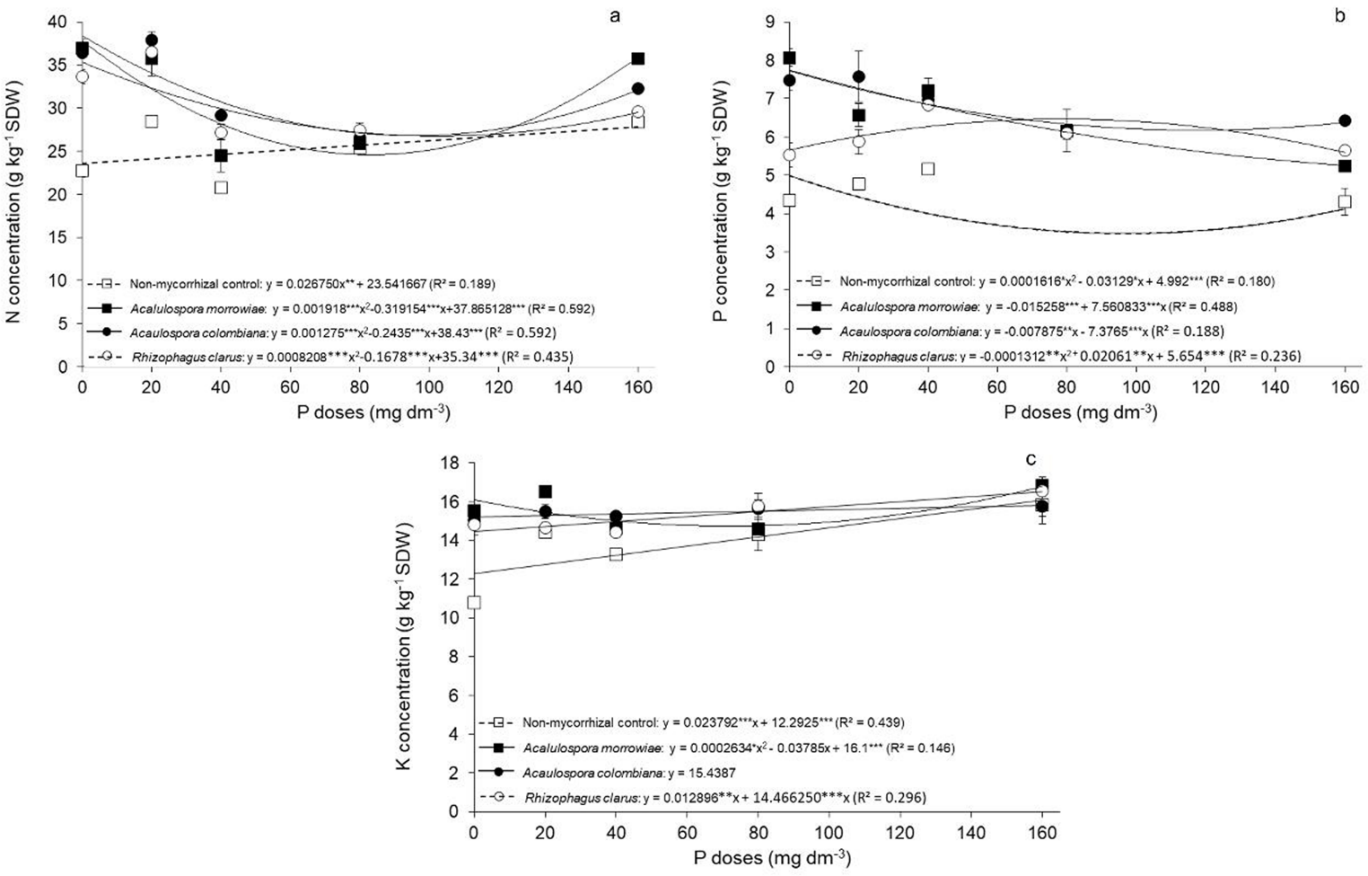
Nitrogen (**a**), Phosphorus (**b**) and Potassium (**c**) concentrations in plant tissue from *Mimosa scabrella* growth with or without arbuscular mycorrhizal fungus *Acaulospora morrowiae*, *Acaulospora colombiana* and *Rhizophagus clarus* cultivated under different P levels. Averages were obtained from 6 replicates and error bars represent the standard error of the mean. The horizontal lines are non-significant regression. Asterisks represent significant differences as calculated by ANOVA (*p < 0.05, **p < 0.01, ***p < 0.001)

The inoculation of AM fungi significantly changed plant shoots nutrient status. The nitrogen concentration exhibited an average increase of 26% with AMF inoculation compared with the non-mycorrhizal treatment. This effect was particularly pronounced at lower doses of phosphorus in which AM fungi increased the N concentration by 57%, 29% and 30% at 0, 20, and 40 mg P dm^− 3^, respectively. Phosphorus concentration was notably influenced by the inoculation of *M. scabrella* with AM fungi even in the high doses of P. At 80 mg P dm^− 3^, plants inoculated with *A. colombiana*, *A. morrowiae* and *R. clarus* had shoot P levels 144%, 146% and 144% higher, respectively, compared to non-mycorrhizal plants. Furthermore, at lower doses of phosphorus supply (0 and 20 mg P dm^− 3^), the phosphorus concentration increased by 28% and 23% for *Rhizophagus clarus*, 72% and 59% for *Acaulospora colombiana*, and 86% and 48% for *Acaulospora morrowiae*, respectively, relative to the non-mycorrhizal control. The potassium concentration was significantly increased by AM fungi inoculation at 20 mg P dm^− 3^ only, increasing 38% for *R clarus*, 40% for *A. colombiana* and 44% for *A. morrowiae*, compared to non-mycorrhizal controls (Fig. 4).

**Fig. 5:**
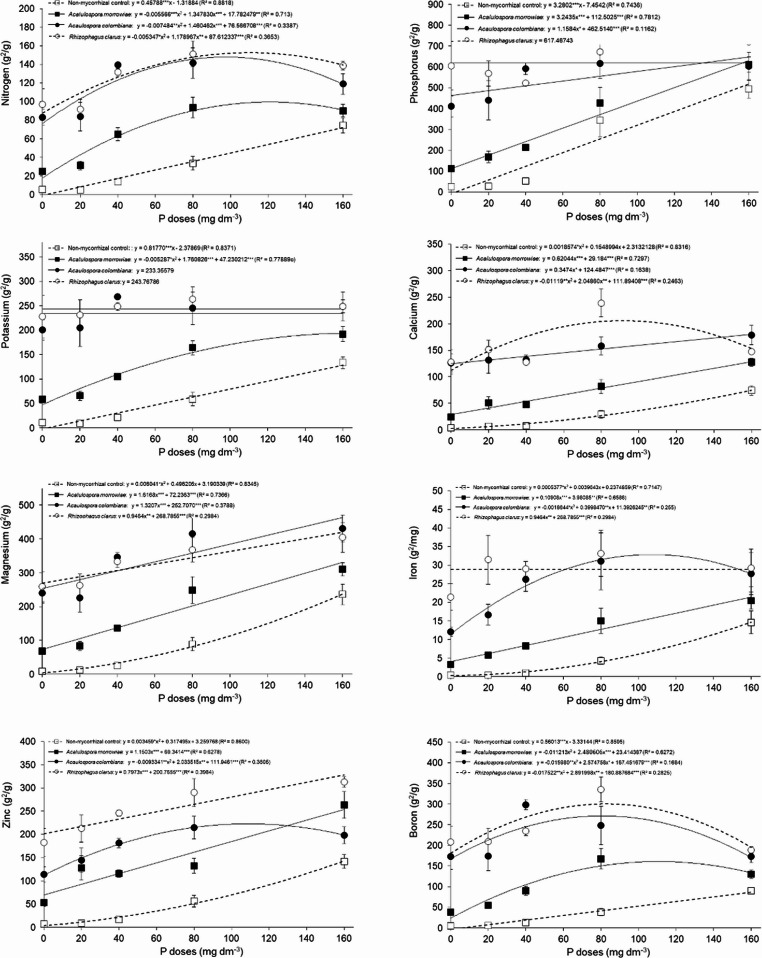
Nutrient use efficiency (NUE) of nutrients from *Mimosa scabrella* seedlings grown with or without AMF *Acaulospora morrowiae*, *Acaulospora colombiana* and *Rhizophagus clarus* cultivated under different P levels. Averages were obtained from 6 replicates and error bars represent the standard error of the mean. Horizontal lines are non-significant regression. Asterisks represent significant differences as calculated by ANOVA (*p < 0.05, **p < 0.01, ***p < 0.001)

Nutrient use efficiency (NUE) for N, P, K, Ca, Mg, Fe, Zn, and B was influenced by mycorrhizal and phosphorus levels. Overall, plants inoculated with *Acaulospora colombiana* (AC), *Rhizophagus clarus* (RC) accumulated more nutrients in plant tissue and exhibited higher NUE compared with those inoculated with *A. morrowiae* (AM) and non-mycorrhizal control for all nutrients (Fig. 5 and Supplementary Material Fig. S2). For instance, averaging overall phosphorus levels and comparing with the non-mycorrhizal control, *R. clarus*,* A. colombiana* and *A. morrowiae* increased NUE by 226, 181 and 63% of phosphorus, 366, 333 and 132% of nitrogen, 422, 400 and 150% of potassium, 555, 500, and 174% of Calcium, 340, 348 and 129% of Magnesium, 598, 450 and 155% of Iron, 442, 272 and 202% of zinc and 677, 604 and 217% of boron, all respectively. Despite this overall increase in NUE in plants inoculated with AM fungi for all P levels, the magnitude of the increase was larger at lower doses. For example, plants inoculated with *A. colombiana*, *A. morrowiae* and *R. clarus* had NUE of 1487, 370 and 1753% for Nitrogen at 0 mg P dm^− 3^ and 60, 21 and 86% at 160 mg P dm^− 3^, respectively.

## Discussion

This study demonstrates that *Mimosa scabrella*, a leguminous tree of significant ecological and economic importance, consistently benefits from its symbiosis with arbuscular mycorrhizal (AM) fungi. Inoculation with three distinct AM fungal isolates significantly enhanced plant growth, nutrient uptake, and nutrient use efficiency, alongside notable alterations in root architecture, particularly under conditions of low phosphorus availability. While these positive effects underscore the potential of AM symbiosis in applications such as sustainable forestry and land restoration, particularly for a tropical pioneer species (Siqueira and Saggin-Junior 2001) like *M. scabrella* and perennial plants are highly dependent on AM fungi (Koziol et al. 2015; Koziol and Bever 2016; Tsiknia et al. 2021), a more refined ecological and physiological interpretation is needed. The observed magnitude of benefits, while substantial, may also involve complex trade-offs in carbon allocation or resource partitioning that are not fully elucidated by growth metrics alone (Bennett and Groten 2022). Future research could explore these underlying mechanisms to provide a more comprehensive understanding of the symbiosis’s ecological costs and benefits.

*M. scabrella* established a functional and beneficial symbiosis with all AM fungal isolates tested (*A. colombiana*,* A. morrowiae*, and *R. clarus*), however, the magnitude of the results varied across fungal isolates. *A. morrowiae* was less effective than *A. colombiana* and *R. clarus* in enhancing plant growth and nutrient use efficiency. Inoculating *M. scabrella* with the same species of AM fungi but different isolates, Stofel et al. (2016) showed that *A. colombiana* was less effective compared to the other two species. Pouyu-Rojas et al. (2006) inoculated AM fungi in 16 woody species and also observed that isolates of *R. clarus* and *A. colombiana* were efficient for a wide range of hosts. These results corroborate that mycorrhizal function is context-dependent and it is influenced by a combination of plant, fungal and soil factors (Hoeksema et al. 2010; Lammel et al. 2015; Primieri et al. 2021) demonstrated for *M. scabrella* synergistic effects of co-inoculation with *Burkholderia* sp., reporting mycorrhizal efficiencies of 246% with *Acaulospora koskei* and 267% with *Rhizophagus intraradices*, respectively, compared to uninoculated plants. Considering that different isolates of both genera *Acaulospora* and *Rhizophagus* performed well under varying experimental conditions, it can be inferred that these fungal genera are generally effective in enhancing the growth and nutrition of woody plants.

Consistent with established ecological principles, the mycorrhizal responsiveness of M. scabrella to the tested AM fungi decreased as phosphorus availability in the soil increased. We define mycorrhizal responsiveness as the difference in plant growth between mycorrhizal and non-mycorrhizal individuals at a given soil phosphorus concentration (Janos 2007). This metric is distinct from mycorrhizal dependence, which refers to the minimum phosphorus level required for a plant to grow in the absence of mycorrhizal fungi (Janos 2007). While our non-mycorrhizal plants exhibited poor growth even at the highest phosphorus levels compared to inoculated plants, suggesting a high degree of reliance on AM fungi, we must exercise caution in inferring true mycorrhizal dependence. The co-inoculation of all treatments with *Burkholderia* spp. complicates the isolation of AMF-specific dependence, as these bacteria also contribute to plant nutrition (Primieri et al. 2021). Therefore, while *M. scabrella* demonstrates high responsiveness to AMF across a range of P levels, a definitive assessment of its mycorrhizal dependence on AMF alone would require experimental designs that decouple the effects of AMF and *Burkholderia* across a full gradient of nutrient availability. Our findings, however, strongly indicate that *M. scabrella* significantly benefits from AMF presence, particularly under phosphorus-limiting conditions.

Plants inoculated with AM fungi are expected to have phosphorus nutrition improved, which could shift the growth limitation to other nutrients, such as nitrogen. In this study, we inoculated seedlings of *M. scabrella* with a strain of *Burkholderia* to prevent nitrogen limitation, as this bacteria fix all nitrogen required for *M. scabrella* growth (Primieri et al. 2016). This dual symbiosis with AM fungi and rhizobia supplies the plant with both of these potentially limiting, complementary nutrients and may enhance the plant’s ability to uptake other nutrients (Wu et al. 2023) and use them more efficiently. Plants with higher NUE have higher growth with relatively lower levels of applied or absorbed nutrients, reducing the cost–benefit of agricultural systems (Siddiqi and Glass 1981; Choudhary et al. 2021). Inoculation of *M. scabrella* with AM fungi, particularly *Rhizophagus clarus* and *Acaulospora colombiana*, significantly increased uptaking of essential macronutrients (N, P, K, Ca, and Mg) and micronutrients (Fe, Zn, and B) compared with non-mycorrhizal plants. This improvement is attributed to the direct effects of the AM fungi external mycelium that has a higher affinity for certain nutrients (Bücking and Kafle 2015) especially in nutrient-poor environments (Pérez-Tienda et al. 2012; Priyadarshini et al. 2022). The quadratic responses observed for N, P, and B suggests an optimal P dose beyond which NUE declines, potentially due to feedback regulation mechanisms that limit excessive nutrient accumulation and maintain homeostasis in mycorrhizal symbioses (Shi et al. 2021; Chiu and Paszkowski 2019).

However, while the observed increases in NUE for multiple nutrients are significant, the interpretation of these indices requires caution. NUE, as calculated in this study, is inherently coupled with total biomass production (Siddiqi and Glass 1981). Consequently, the higher NUE values in mycorrhizal plants may partially reflect a “size effect” where larger, faster-growing plants appear more efficient simply due to their greater capacity for biomass accumulation rather than a fundamental shift in physiological utilization efficiency (Stahlhut et al. 2024). Future studies should utilize allometric analysis or specific utilization rates to decouple the influence of plant size from intrinsic physiological efficiency, providing a more robust assessment of the symbiosis’s role in nutrient economy.

Our study demonstrated that inoculation with AM fungi significantly altered the root architecture of *M. scabrella* seedlings compared to non-mycorrhizal controls, increasing parameters such as average root diameter, total length, surface area, and volume. Conversely, specific root length (SRL), a key index of soil exploration efficiency (Ostonen et al. 2007), decreased in mycorrhizal plants across all P levels. While it is plausible to interpret this reduction in SRL and increase in diameter as a functional replacement of root foraging by the extensive fungal hyphal network (Smith and Read 2008), allowing the plant to reallocate photosynthates to above-ground growth (Chen et al. 2016; Bennett and Groten 2022), this interpretation must be approached with caution. The data presented do not directly confirm such functional replacement. Alternative explanations, such as allometric effects driven by the significantly larger size of mycorrhizal plants, should be considered (Maherali 2014; Zhang et al. 2011). The strong correlation observed between total plant biomass and root traits (r² = 0.56; *p* < 0.001) suggests that some of these architectural shifts may be size-dependent rather than solely symbiosis-specific adaptations. Indeed, a lack of control for allometric scaling between root architecture and plant size can confound interpretations of root plasticity in response to fungal colonization (Maherali 2014). Furthermore, changes in carbon allocation unrelated to foraging efficiency could also contribute to the observed morphology. Future studies should aim to disentangle these allometric and physiological factors to better understand the mechanisms driving root architectural responses in mycorrhizal symbioses.

Despite the changes in root architecture, mycorrhizal root colonization was not negatively affected by phosphate fertilization. In fact, higher levels of root colonization (> 90%) by all three fungal isolates were detected at 160 mg P dm⁻³ treatment. This result contradicts general pattern reported in the literature that increases in P availability generally reduces mycorrhizal colonization, as plants tend to rely less on the symbiosis for phosphorus (Lambers et al. 2018; Janos 2007). The maintenance of high mycorrhizal colonization at the highest P level is noteworthy, as elevated P availability typically triggers a downregulation of the symbiosis to avoid unnecessary carbon costs (Balzergue et al. 2013). This persistence suggests that the fungal isolates, particularly *Rhizophagus clarus*, may exhibit a “luxury colonization” strategy or that the high P demand of *M. scabrella*—potentially amplified by the metabolic requirements of the *Burkholderia* co-symbiosis—prevents the typical P-induced inhibition of AMF (Wang et al. 2026). However, high colonization under these conditions does not unequivocally imply continued mutualistic benefit. As suggested by (Smith et al. 2011), the functional significance of the mycorrhizal uptake pathway can be decoupled from colonization levels, potentially leading to “growth depressions” if carbon costs exceed nutrient gains. Without direct quantification of the mycorrhizal P-uptake pathway versus the direct root pathway, the net benefit at high P remains speculative.

Considering that root length and root surface tended to increase in mycorrhizal treatments compared to control in all P levels, our results suggest that fungal isolates used herein have the ability to initiate root colonization fast and produce extensive internal mycelium. It is interesting that *R. clarus* extensively colonized *M. scabrella* roots regardless of P levels, supporting the suggestions that fungi in Glomeraceae, particularly *Rhizophagus* species, are more efficient at colonizing roots and maintaining symbiotic relationships under varying soil conditions (Smith and Read 2008). *R. clarus* has been reported worldwide and adapted to a wide range of climatic and soil conditions, including high levels of soil P (Stürmer et al. 2025) and our results suggest that this species has a great potential to be included into mycorrhizal commercial inoculants. The high mycorrhizal root colonization was correlated with high biomass production of *M. scabrella* (r^2^ = 0.36; *p* < 0.001; Fig. S3) across all phosphorus levels and fungal isolates, a general tendency already demonstrated by meta-analysis (Treseder 2013). Production of seedlings with roots extensively colonized by mycorrhizal fungi is important to improve growth and survival of plants when transplanted to field conditions.

The findings from *M. scabrella* contribute to a broader understanding of mycorrhizal systems across both natural and managed ecosystems. In agricultural systems, such as common bean (*Phaseolus vulgaris*), the interaction between AMF, rhizobia, and phosphorus availability plays a central role in optimizing plant performance under variable environmental conditions (Rodiño et al. 2025). Furthermore, recent evidence indicates that combining AMF with soil amendments such as biochar can enhance plant growth and soil functionality under stress conditions (Jabborova et al. 2025). However, these systems differ substantially from the present study in terms of plant life history, environmental constraints, and soil management practices. While biochar-mediated improvements are often linked to changes in soil structure, nutrient retention, and microbial habitat, our results highlight strong mycorrhizal responsiveness under controlled conditions without such amendments. Therefore, although these findings collectively emphasize the importance of managing mycorrhizal symbioses, caution is needed when extrapolating across systems, as the magnitude and mechanisms of AMF benefits are likely context-dependent.

## Conclusion

This study confirms that *M. scabrella* establish a functional mycorrhizal association with AM fungi, regardless of the soil phosphorus levels. Inoculation with three isolates of AM fungi and a strain of *Burkolderia* significantly improved biomass accumulation, nutrient uptake and use efficiency, and changed root architecture parameters. While inoculation with *Acaulospora colombiana* and *Rhizophagus clarus* was slightly more effective than with *Acaulospora morrowiae*, all fungi extensively colonized roots of *M. scabrella* regardless of soil P levels. These findings reinforce the importance of selecting highly efficient AM fungi species (or isolates) for agricultural and ecological applications, especially in soils with varying phosphorus levels. Further studies should investigate the functional differences between these fungal species to optimize their use in sustainable practices.

## Supplementary Information

Below is the link to the electronic supplementary material.

Supplementary Material 1

## Data Availability

No datasets were generated or analysed during the current study.
