# Supplementary material for: Arbuscular mycorrhizal fungi change root morphology and nutrient use efficiency in the tree legume Mimosa scabrella
Source: Mycorrhiza. 2026 Apr 14;36(2):15. doi: 10.1007/s00572-026-01260-9 (PMC13076420; doi:10.1007/s00572-026-01260-9)
Supplement: Supplementary file 1 — Supplementary Material 1 [file 572_2026_1260_MOESM1_ESM.docx]

**Arbuscular mycorrhizal fungi change root morphology and nutrient use efficiency in the tree legume *Mimosa scabrella***

**Author list**

Silmar Primieri^1^, Murilo Dalla Costa^2^, Tássio Dresch Rech^2^, Marlise Nara Ciotta^2^, Sidney L. Stürmer^3^

*^1^Campus Lages, Federal Institute of Education, Science and Technology of Santa Catarina (IFSC), Lages, SC 88509-640, Brazil*

*^2^Experiment Station of Lages, Santa Catarina State Agricultural Research and Rural Extension Agency (EPAGRI), P.O. Box 181, Lages, SC 88502-970, Brazil*

*^3^ Departamento de Ciências Naturais, Universidade Regional de Blumenau, R. Antônio da Veiga 140, Blumenau, Santa Catarina 89030-903, Brazil*

**Author for correspondence details**

Silmar Primieri: [silmar.primieri@ifsc.edu.br](mailto:silmar.primieri@ifsc.edu.br)

**Table S1** Correlation matrices for root architecture responses and total dry biomass

| Treatment | Root Length  (m plant^-1^) | Proj Area  (cm^2^ plant^-1^) | Surface Area  (cm^2^ plant^-1^) | Root Volume  (cm^3^ plant^-1^) | Number of Tips | Root dry biomass  (g plant^-1^) | Shoot dry biomass  (g plant^-1^) |
| --- | --- | --- | --- | --- | --- | --- | --- |
| Root Length  (m plant^-1^) | 1 | 0.97 | 0.97 | 0.88 | 0.81 | 0.84 | 0.74 |
| Proj Area  (cm^2^ plant^-1^) |  | 1 | 1 | 0.97 | 0.75 | 0.9 | 0.79 |
| Surface Area  (cm^2^ plant^-1^) |  |  | 1 | 0.97 | 0.97 | 0.9 | 0.79 |
| Root Volume  (cm^3^ plant^-1^) |  |  |  | 1 | 0.65 | 0.91 | 0.79 |
| Number of Tips |  |  |  |  | 1 | 0.64 | 0.58 |
| Root dry biomass |  |  |  |  |  | 1 | 0.79 |
| Shoot dry biomass |  |  |  |  |  |  | 1 |


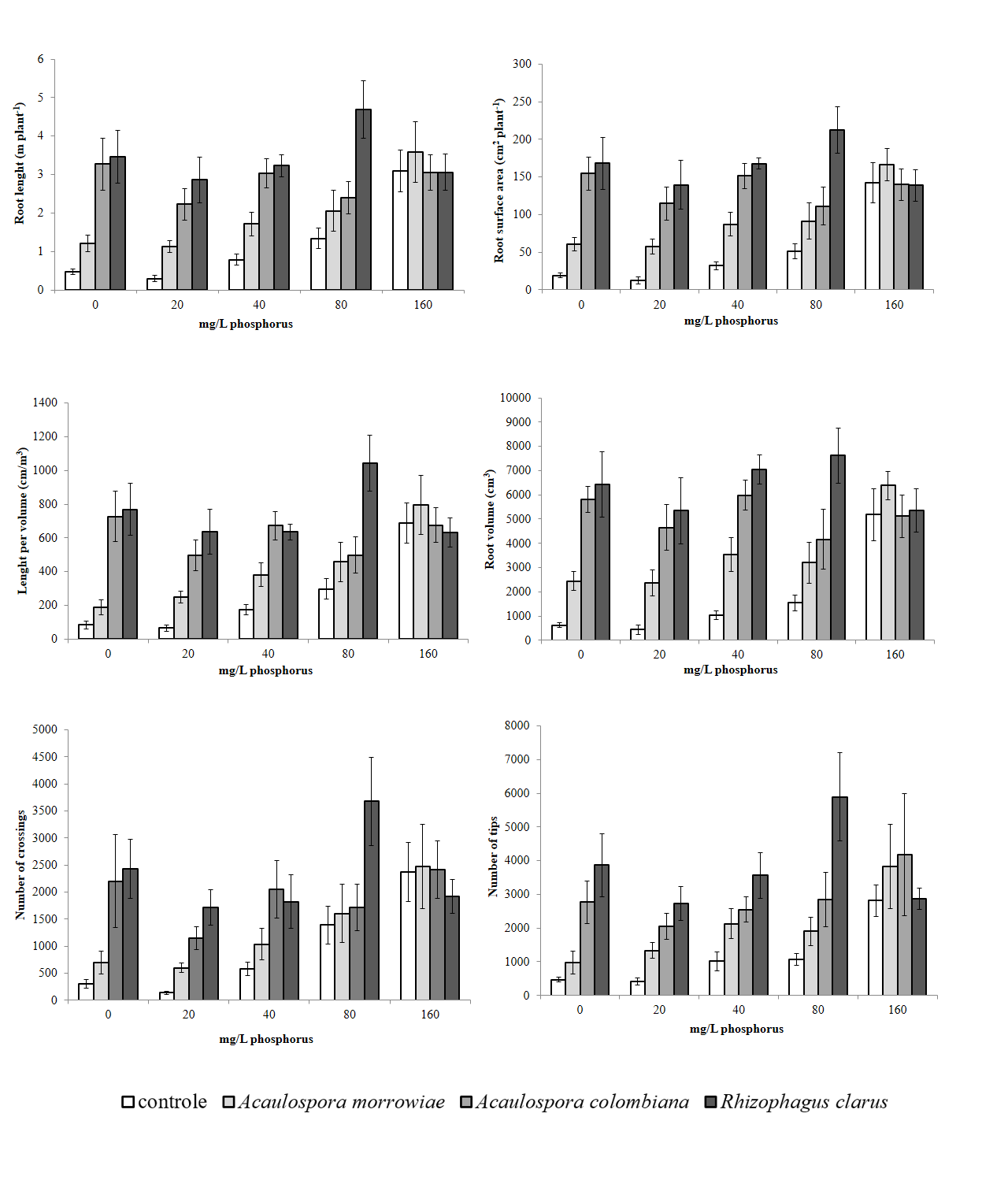


**Fig. S1** Root architecture responses of *Mimosa scabrella* roots growth with or without arbuscular mycorrhizal fungus *Acaulospora* *morrowiae*, *Acaulospora colombiana* and *Rhizophagus clarus* cultivated under different P levels. Bar represent means (n=6) and error bars are the standard error. Bar represent means (n=6) and error bars are the standard error.


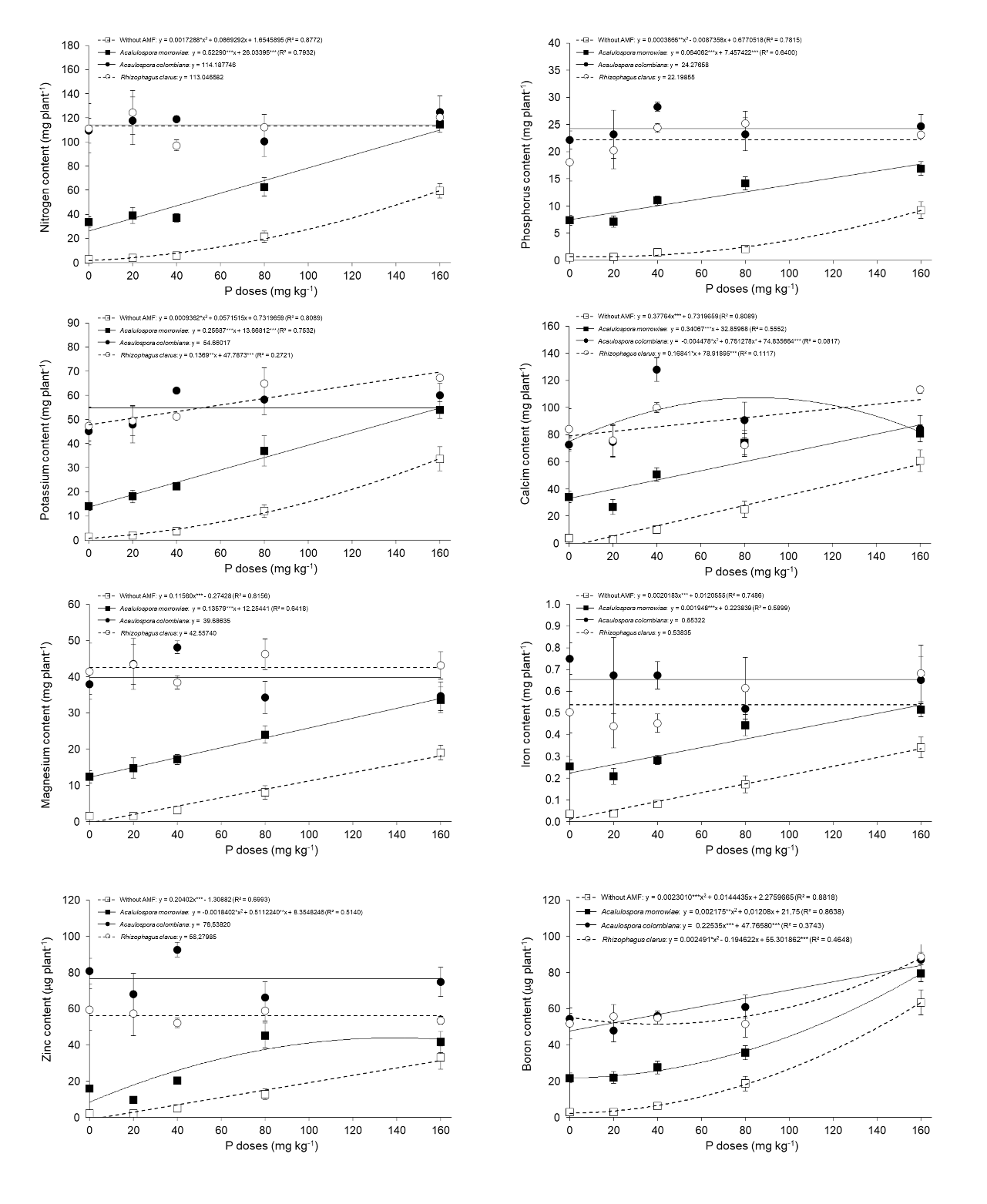


**Fig. S2** Nutrient content of nutrients from *Mimosa scabrella* seedlings growth with or without AMF *Acaulospora morrowiae*, *Acaulospora colombiana* and *Rhizophagus clarus* cultivated under different P levels. Averages were obtained from 6 replicates and error bars are the standard error of the mean. Horizontal lines are non-significant regression.


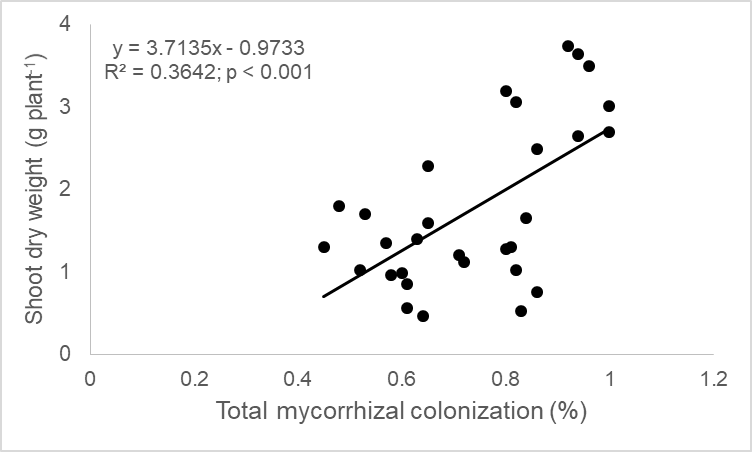


**Fig. S3** Correlation between Shoot dry weight (g plant^-1^) and total mycorrhizal colonization (%) of *Mimosa scabrella* roots inoculated with *Acaulospora morrowiae, Acaulospora colombiana* and *Rhizophagus clarus*
